# Evaluation of Electrolytes Imbalance and Dyslipidemia in Diabetic Patients

**DOI:** 10.3390/medsci4020007

**Published:** 2016-04-01

**Authors:** Nabil A. Hasona, Abdulbaset Elasbali

**Affiliations:** 1 Department of Biochemistry, College of Medicine, University of Hail, PO Box 2440 Hail, Saudi Arabia; 2 Faculty of Science, Chemistry Department, Biochemistry division, Beni-Suef University, Beni-Suef, Egypt; 3 College of Applied Medical Science, Clinical laboratory Department, Hail University PO Box 2440 Hail, Saudi Arabia; elasbali2000@hotmail.co.uk

**Keywords:** electrolytes, diabetic, Na^+^, K^+^, Ca^++^, lipid

## Abstract

Electrolytes and Lipids have always played significant roles, and changes in their concentrations gives good indications of disease progression in a number of non-communicable diseases. Diabetes mellitus is the most common metabolic disorder in the community. Diabetics may suffer from electrolyte disorders due to complications of diabetes mellitus and the medication they receive. Serum glucose, electrolytes (Na^+^, K^+^, Cl^−^ and Ca^++^), and lipid profiles (total cholesterol, triglyceride, and HDL-c) were determined in 100 diabetics and in non-diabetic subjects. All the diabetic patients had a significant (*p* < 0.001) increase in glucose, total cholesterol, triglyceride, chloride and calcium levels. There was significant (*p* < 0.001) decrease in the serum levels of Na^+^ and K^+^ in all diabetics. It was concluded that differences in lipids and electrolytes found in diabetics may have great potential as a diagnostic tool in clinical practice and have a significant effect upon the risk of contracting many diseases.

## 1. Introduction

Electrolytes are present in the human body. Electrolytes play an important role in many body processes, such as controlling fluid levels, acid-base balance (pH), nerve conduction, blood clotting and muscle contraction. Potassium, sodium and calcium are all important for proper electrolyte balance. Electrolyte imbalance resulting from kidney failure, dehydration, fever, and vomiting has been suggested as one of the contributing factors toward complications observed in diabetes and other endocrine disorders [1].

Diabetes mellitus is a heterogeneous group of metabolic disorder characterized by high blood glucose level (hyperglycemia) with alteration in carbohydrate, lipid, and protein metabolism resulting from defects in insulin secretion and/or action [2].

The main symptoms of diabetes include increased urination, increased thirst, fatigue, weight loss, blurred vision, increased hunger, and diabetic dermatomes. Any test used to diagnose diabetes requires confirmation with a second measurement unless clear symptoms of diabetes exist.

There are probably 100 million people in the world with diabetes mellitus and incidences of diabetes are on the rise. As diabetes progresses, patients are at increased risk of developing the coronary disease [3].

Hyperglycemia sets the internal environment for osmotic diuresis while causing a dilutional effect on electrolyte concentrations. The osmotic effect of glucose results in decreased circulating blood volume and fluid shift from the intracellular spaces causing cellular dehydration. This study aims to identify the relationship of serum electrolytes (sodium, calcium, chloride, and potassium) and lipid profile with fasting blood glucose levels in diabetic subjects. We selected sodium, potassium, calcium, and chloride because those are the most common macro electrolytes and correlated with Diabetes mellitus.

## 2. Patients and Methods

The study was carried out at the College of Applied Medical Sciences, Hail University. A total of 100 subjects were included in the study, out of which two groups were formed: 50 (25 male and 25 female) type 2 diabetes patients and 50 (20 female and 30 male) controls. According to the American Diabetes Association, patients that have fasting glucose ≥7 mmol/L will be diabetic. Regarding the age group, the majority of our patients were in the age group ranging from 36–70 years. Patients who were diagnosed mainly with type 2 DM less than 3 years ago and those with conditions like renal disease, other chronic illness, alcohol intake, and pregnancy were excluded from the study.

## 3. Ethical Consideration

The objective of the study was well explained to all participants in this study. The anonymity of patients was maintained by coding the sample. Permission for this study was obtained from the college ethical committee.

## 4. Collection of Sample

Blood samples were collected and serum vacutainers in the morning after 12 h of fasting and the serum was separated within 30–45 min, aliquoted and stored at −20 °C for further analysis. The study was conducted at Biochemistry lab, University of Hail, Hail, KSA.

## 5. Analytical Methods

Serum analysis for fasting glucose, Na^+^, K^+^, Ca^++^ and Cl^−^ was performed by the automatic analyzer, ROCHE module Cobas 6000 (C-501 and C-601), and kits were procured by ROCHE. Serum total cholesterol levels was determined by enzymatic (CHOD-PAP) colorimetric method [4] and triglycerides by enzymatic (GPO-PAP) method of [5]. HDL-cholesterol was estimated using precipitant method [6].

Statistical analyzes were done using the Statistical Package for the Social Sciences (SPSS for windows, version 13.0; SPSS Inc., Chicago, IL, USA). Data were presented using mean ± standard error mean (M ± SEM) for all quantitative values. Statistical significance was determined as a *p* value < 0.01 were considered statistically highly significant and a *p* value < 0.001 were considered statistically very highly significant.

## 6. Results

A total of 100 patients were included in this study (45 F, 55 M), with an age range from 36 to 70 years. They were divided into two groups depending on the serum glucose level. The first group consisted of 50 patients (25 F, 25 M), who had serum glucose below 6 mmol/L (normal control). The second group consisted of 50 patients (20 F, 30 M), with serum glucose more than 7 mmol/L (diabetic subjects).

The diabetic subjects exhibited a very highly significant (*p* < 0.001) increase in serum fasting blood glucose, total cholesterol and triglycerides levels. In contrast, HDL-C level for diabetic patients showed a very highly significant (*p* < 0.001) decrease (Table 1).

On the other hand, we selected sodium, potassium, calcium, and chloride because those are the most common macro electrolytes and correlated with Diabetes mellitus. The serum electrolytes level (Na^+^ and K^+^) for diabetic subjects showed a very highly significant (*p* < 0.001) decrease. In contrast, serum Ca^++^ and Cl^−^ levels exhibited a very highly significant (*p* < 0.001) increase (Table 2).

## 7. Discussion

Derangement of water and electrolyte balances may occur in subjects with diabetes mellitus, resulting from insulin deficiency, hyperglycemia, and hyperketonemia [2]. The present study showed a very highly significant reduction in serum Na^+^ and K^+^ levels and an elevation in serum Ca^2+^ and Cl^−^ in subjects with DM were observed. This result was consistent with those reported by previous studies [7,8].

Under physiological conditions, most of the Na^+^ is reabsorbed in the proximal tubule of the kidney [9].

Hyperglycemia is restricted to the extracellular space so water moves from the intracellular to the extracellular compartment initially, diluting plasma sodium. During the accompanying osmotic diuresis, water is generally lost in excess of sodium until eventually the loss of water is similar to both extracellular and intracellular compartments. Therefore, in diabetes mellitus, plasma sodium concentrations may be artificially lowered. Hyperglycemia-induced osmotic diuresis which can increase excretion is thought to be a primary mechanism underlying the decreased serum concentrations of Na^+^ observed in response to elevated glucose levels.

Ca^2+^ is mainly reabsorbed in the proximal tubule. Its reabsorption is coupled to Na^+^ absorption. In the distal convoluted tubule, Ca^2+^ absorption is regulated independently of Na^+^, where numerous factors, such as calcitonin, parathyroid hormone, and vitamin D, can have marked effects on Ca^2+^ reabsorption and secretion [9].

On the other hand, the serum potassium levels were reduced because of diuretics and the diabetic ketoacidosis (increased loss in the urine). 

It has been observed that cellular membrane electrolyte transporter Na^+^-K^+^-ATPase dysfunction in diabetic subjects can be secondary to hyperglycemia [10]. Moreover, the functions of Ca^2+^-Mg^2+^-ATPase, Na^+^/Ca^2+^ exchanger, and Ca^2+^ pump, which are located in the cell membrane, mitochondria or endoplasmic reticulum, have been shown to be impaired in diabetes [11].

Regarding the lipid profile, the present results revealed a significant increase (*p* < 0.001) in the serum levels of cholesterol and triglycerides and a significant decrease in HDL-cholesterol in all diabetics relative to all non-diabetic subjects. These results are in agreement with Lamarche *et al.* [12].

In diabetes, many factors may affect blood lipid levels causing dyslipidemia, the most important of which probably is insulin deficiency, which plays an important role in the regulation of intermediary metabolism. Also, Hyperglycemia progressively increases the transfer of cholesterol esters from HDL-C to VLDL-C particles [13].

The relative insulin deficiency that occurs in type 2 diabetes impairs the action of lipoprotein lipase and results in lower HDL-C levels and higher TG levels, which may improve with improved glycemic control [14].

## 8. Conclusions

It was concluded that differences in lipids and electrolytes found in diabetics may have a great potential as a diagnostic tool in clinical practice. Electrolyte imbalance has a significant effect upon the risk of contracting many diseases. Also, early diagnosis, good glycemic control, and dietary modification are usually enough for prevention and treating complications in diabetes mellitus.

## 9. Limitations of the Study

Anthropometric and blood pressure data for the patients were not obtained.

## Figures and Tables

**Table 1:** levels of serum glucose and lipid profile in diabetic and control subjects.

Parameters	Normal Control Subjects	Diabetic Treated Subjects
Fasting glucose (mmol/L)	4.96 ± 0.06	9.52 ± 0.20 ***
Cholesterol(mmol/L)	4.00 ± 0.10	6.24 ± 0.10 ***
Triglycerides(mmol/L)	1.26 ± 0.07	3.25 ± 0.1 ***
HDL-C(mmol/L)	1.18 ± 0.02	0.76 ± 0.02 ***

Values are expressed as means ± SEM, *** *p* < 0.001 as compared to normal control subjects.

**Table 2:** levels of serum electrolyte profile in diabetic and control subjects.

Parameters	Normal Control Subjects	Diabetic Treated Subjects
Na^+^ (mmol/L)	138.88 ± 0.19	133.72 ± 0.31 ***
K^+^ (mmol/L)	4.53 ± 0.06	4.17 ± 0.062 ***
Cl^−^ (mmol/L)	102.56 ± 0.20	104.54 ± 0.24 ***
Ca^++^ (mmol/L)	2.20 ± 0.02	2.37 ± 0.01 ***

Values are expressed as means ± SEM, *** *p* < 0.001 as compared to normal control subjects.
