# Correction: Hasona, N.A. and Elasbali, A. Evaluation of Electrolytes Imbalance and Dyslipidemia in Diabetic Patients. *Med. Sci.* 2016, *4*, 7

**DOI:** 10.3390/medsci5010004

**Published:** 2017-01-22

**Authors:** Nabil A. Hasona, Abdulbaset Elasbali

**Affiliations:** 1 Department of Biochemistry, College of Medicine, University of Hail, PO Box 2440 Hail, Saudi Arabia; 2 Faculty of Science, Chemistry Department, Biochemistry Division, Beni-Suef University, Beni-Suef 62514, Egypt; 3 College of Applied Medical Science, Clinical Laboratory Department, Hail University PO Box 2440 Hail, Saudi Arabia; elasbali2000@hotmail.co.uk

The authors wish to make the following correction to their paper [1]. In the discussion section, the sentence “Regarding the lipid profile, the present results revealed a significant increase (*p* < 0.001) in the serum levels of cholesterol, triglycerides and HDL-cholesterol in all diabetics relative to all non-diabetic subjects”. This should be changed to “Regarding the lipid profile, the present results revealed a significant increase (*p* < 0.001) in the serum levels of cholesterol and triglycerides and a significant decrease in HDL-cholesterol in all diabetics relative to all non-diabetic subjects”.

The authors would like to apologize for any inconvenience caused to the readers by these changes. The manuscript will be updated and the original will remain online on the article webpage.
